# Supplementary material for: 2D-DIGE-MS Proteomics Approaches for Identification of Gelsolin and Peroxiredoxin 4 with Lymph Node Metastasis in Colorectal Cancer
Source: Cancers (Basel). 2022 Jun 29;14(13):3189. doi: 10.3390/cancers14133189 (PMC9265116; doi:10.3390/cancers14133189)
Supplement: Supplementary file 1 [file cancers-14-03189-s001.zip › File S1.pdf]

Figure 4A

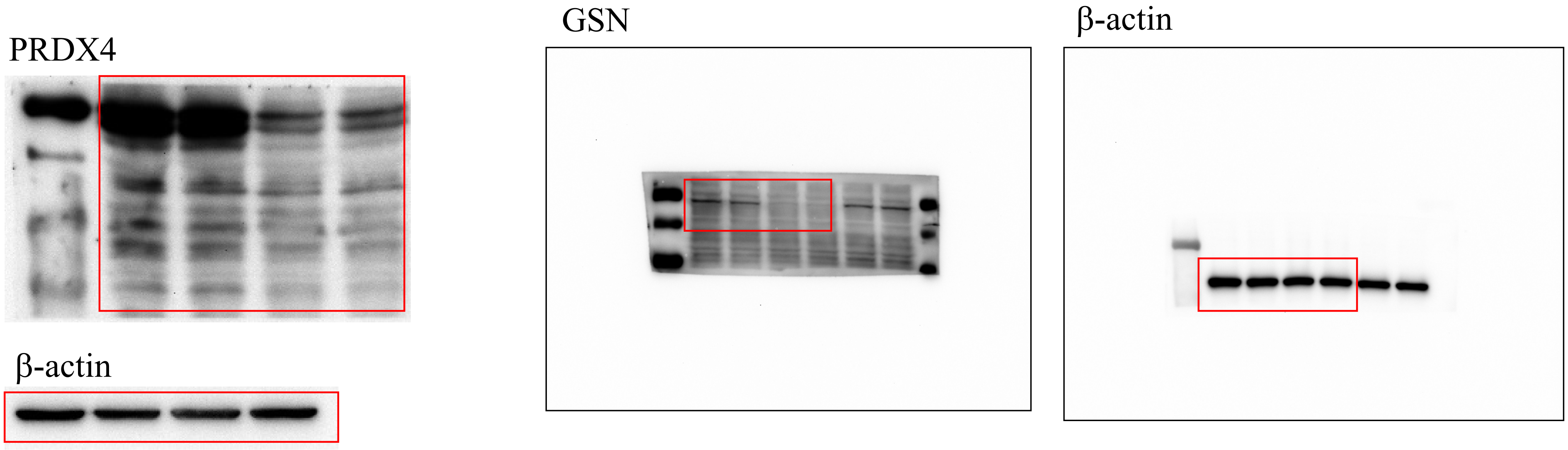

Figure 6

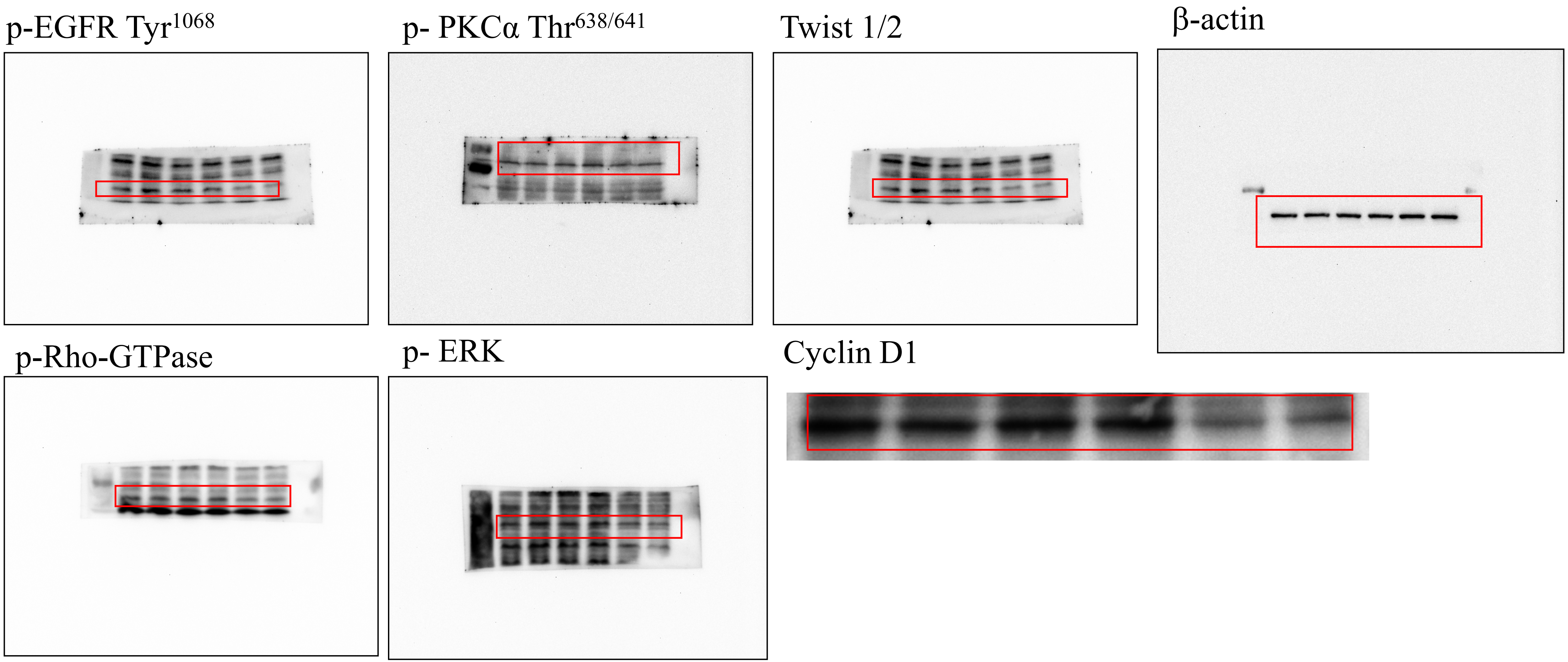
p-EGFR Tyr1068
p- PKCα Thr638/641
Twist 1/2
β-actin
p-Rho-GTPase
p- ERK
Cyclin D1
