# Supplementary material for: 2D-DIGE-MS Proteomics Approaches for Identification of Gelsolin and Peroxiredoxin 4 with Lymph Node Metastasis in Colorectal Cancer
Source: Cancers (Basel). 2022 Jun 29;14(13):3189. doi: 10.3390/cancers14133189 (PMC9265116; doi:10.3390/cancers14133189)
Supplement: Supplementary file 1 [file cancers-14-03189-s001.zip › Table S1.pdf]

cancers MDPI

Table S1. Baseline characteristics of patients with colorectal cancer.

Baseline characteristics	Cases
Average age	64.8 (27-89)
**Gender**	
Male	21
Female	19
Average tumor diameter (cm)	4.2
**Tumor location**	
Colon	28
Rectum	12
**TNM staging**	
I + II stage	18
III + IV stage	22
**Differentiation degree**	
Moderately or poorly differentiated	29
Highly differentiated	11
